# Periradicular repair after single- and two-visit root canal treatments using ultrasonic irrigant activation and calcium hydroxide dressing of teeth with apical periodontitis: study protocol for randomized controlled trials

**DOI:** 10.1186/s13063-022-07030-0

**Published:** 2023-01-12

**Authors:** Gustavo M. Almeida, Vitor Hugo M. Carvalho, Érika B. P. Silva, Marco Antonio F. Cançado, Leonardo S. Barroso, Erica L. Queiroz, Tien Li An, Ana Paula D. Ribeiro, Jacy R. Carvalho-Junior, André F. Leite

**Affiliations:** 1 Postgraduate Program in Health Sciences, College of Health Sciences, University of Brasilia, Brasilia, Brazil; 2 Dental Trauma Project of Department of Dentistry, College of Health Sciences, University of Brasilia, Brasilia, Brazil; 3 Postgraduate Program in Endodontics, University of Taubaté, Taubaté, Brazil; 4 Department of Cariology and Comprehensive Care, New York University College of Dentistry, New York, USA; 5 Department of Dentistry, College of Health Sciences, University of Brasilia, Brasilia, Brazil; 6 Department of Restorative Dental Sciences, Division of Operative Dentistry, College of Dentistry, University of Florida, Gainesville, USA

**Keywords:** Randomized controlled trial, Endodontics, Root canal therapy, Single-visit root canal treatment, Periradicular repair, Apical periodontitis

## Abstract

**Background:**

In certain clinical situations, root canal treatment in teeth with apical periodontitis is performed in multiple visits, with the use of intracanal dressing between visits, aiming to reduce microorganisms and their by-products of the root canal system prior to filling. However, in recent years, discussions have been growing about the real need for the use of intracanal dressing in these cases. The use of ultrasonic activation of the auxiliary chemical substance has increased the potential for decontamination promoted during the chemomechanical preparation of the root canal. Thus, this study is designed to explore whether the use of intracanal dressing between visits during endodontic treatment favors periradicular repair in teeth with apical periodontitis.

**Methods:**

This is a randomized, prospective, double-blinded, controlled clinical trial designed to evaluate 3 distinct clinical approaches used during endodontic therapy: group 1—root canal treatment in a single visit (RCT-SV); group 2—root canal treatment in two visits with intracanal dressing (RCT-TVWD); and group 3—root canal treatment in two visits without intracanal dressing (RCT-TVWOD). A total of 150 adult patients aged 18 to 60, with at least one tooth diagnosed with asymptomatic apical periodontitis and periradicular lesion (confirmed with a cone beam computed tomography (CBCT)), will be randomized and will undergo one of the types of clinical approaches during endodontic therapy. Patients’ postoperative pain levels will also be recorded in periods of 24, 48, and 72 h and 7 days. Subsequently, clinical findings and long-term follow-up evaluations, with periradicular repair, will be performed at 6 and 12 months by intraoral periapical radiograph (IOPAR) and CBCT at the 24-month follow-up.

**Discussion:**

This study will evaluate the periradicular repair of mandibular molar teeth with apical periodontitis, providing information about the efficacy, benefits, and safety of performing the endodontic treatment in a single and two visits, with and without the use of calcium hydroxide dressing. All endodontic therapy procedures will be performed under a dental operating microscope and using ultrasonic activation of auxiliary chemical substances. These results may contribute to changes in the clinical approaches adopted during endodontic therapy of teeth with apical periodontitis and reveal the potential of complementary approaches that aim to enhance the decontamination of the root canal system during the preparation stage.

**Trial registration:**

ClinicalTrials.gov NCT05256667. Registered on 24 February 2022

## Administrative information

The protocol will be reported according to the Standard Protocol Items: Recommendations for Interventional Trials (SPIRIT) statement.

Note: the numbers in curly brackets in this protocol refer to SPIRIT checklist item numbers. The order of the items has been modified to group similar items (see http://www.equator-network.org/reporting-guidelines/spirit-2013-statement-defining-standard-protocol-items-for-clinical-trials/).

**Table:** 

Title {1}	PERIRADICULAR REPAIR AFTER SINGLE- AND TWO-VISIT ROOT CANAL TREATMENTS USING ULTRASONIC IRRIGANT ACTIVATION AND CALCIUM HYDROXIDE DRESSING OF TEETH WITH APICAL PERIODONTITIS: STUDY PROTOCOL FOR RANDOMIZED CONTROLLED TRIALS
Trial registration {2a and 2b}.	ClinicalTrials.gov; identifier NCT05256667. Registered on 24 February 2022
Protocol version {3}	Protocol version 4.0 (November 2022)
Funding {4}	Study funded by authors.
Author details {5a}	Gustavo M. Almeida^1^*, Vitor Hugo M. Carvalho^1^, Érika B. P. Silva^2^, Marco Antonio F. Cançado^2^, Leonardo S. Barroso^3^, Erica L. Queiroz^4^, Tien Li An^5^, Ana Paula D. Ribeiro^6^, Jacy R. Carvalho-Junior^2, 5^, André F. Leite^1, 5^ ^1^Postgraduate Program in Health Sciences, College of Health Sciences, University of Brasilia, Brasilia, Brazil. ^2^Dental Trauma Project of Department of Dentistry, College of Health Sciences, University of Brasilia, Brasilia, Brazil. ^3^Postgraduate Program in Endodontics, University of Taubaté, Taubaté, Brazil. ^4^Department of Cariology and Comprehensive Care, New York University College of Dentistry, New York, USA. ^5^Department of Dentistry, College of Health Sciences, University of Brasilia, Brasilia, Brazil. ^6^Department of Restorative Dental Sciences, Division of Operative Dentistry, College of Dentistry, University of Florida, Gainesville, USA.
Name and contact information for the trial sponsor {5b}	No funding for this study was secured so far. The study was so far funded by the authors and their institutions. Postgraduate Program in Health Sciences College of Health Sciences, University of Brasilia - UnB Campus Universitário Darcy Ribeiro Asa Norte, Brasília, DF, CEP 70910-900, Brazil Phone: +55 61 3107-1753
Role of sponsor {5c}	drgustavoalmeida01@gmail.com Authors are in charge of design; collection, management, writing of the report; and the decision to submit the report for publication, including ultimate authority over any of these activities.

## Introduction

### Background and rationale {6a}

Endodontic therapy in teeth with apical periodontitis is premised on the elimination and inactivation of the greatest possible number of microorganisms within the root canal system through an efficient chemomechanical preparation and three-dimensional filling, allowing the repair of periapical tissues [1]. In cases of apical periodontitis, it is recommended that the efficient chemomechanical preparation should be able to promote cleaning, decontamination, and shaping, being aided by the use of intracanal dressing [2, 3]. Mechanical instrumentation plays a fundamental role in reducing the intracanal microbial load; however, around 30 to 40% of the walls of the main canal are not touched by the instruments [4], thus requiring the irrigating substance to perform an auxiliary chemical action during the instrumentation step [5]. 5.25% sodium hypochlorite (NaOCl) solution has shown satisfactory results due to its excellent properties, such as antimicrobial action, tissue dissolution, bleaching action, and debris removal [6]. Currently, the use of ultrasound as an irrigation intensifying agent associated with a 5.25% NaOCl solution on the root canal walls has been widely discussed [7, 8]. The transformation of electrical energy into mechanical energy using ultrasonic insertion inside the root canal causes a phenomenon in the irrigating solution called “cavitation,” which favors the cleaning and decontamination process of the root canal [9, 10]. Aiming to promote a complementary chemical action to the chemomechanical preparation of root canals, the association of calcium hydroxide [Ca(OH)2] with camphorated paramonochlorophenol (CMCP) and glycerin presents itself as an effective proposal for intracanal dressing to be used between clinical visits so that the dressing helps in the decontamination process, due to its resulting antimicrobial activity. In vitro studies have shown that the Ca(OH)2 paste containing CMCP has a broad antimicrobial spectrum eliminating microorganisms that are resistant to Ca(OH)2 and a wider range of antimicrobial action (eliminating microorganisms located in the most distant regions of the vicinity where the paste medicated was applied) and eliminates microorganisms more quickly than other pastes containing Ca(OH)2 associated only with inert vehicles such as water, saline solution, and glycerin [3, 11, 12].

In the current literature, there are several studies that compared endodontic treatments conducted in a single visit and in multiple visits in teeth with apical periodontitis or in similar conditions [1, 13–18], but the results found are divergent. There is a superiority of results for endodontic treatments conducted in multiple visits with the use of Ca(OH)2-based intracanal dressing in an animal model [13, 14] and in vitro studies [15]. In the most recent systematic reviews [16–18] and in randomized clinical trials [1, 19, 20], the results found are similar to each other in terms of success rate and postoperative pain. This divergence in results may be related to the use of different instrumentation techniques, irrigation, and intracanal dressing protocols in each of these studies, which makes a real comparison between them more difficult. Thus, the search for an efficient protocol for instrumentation and irrigation of root canals and a definition of the need for the use of Ca(OH)2-based intracanal dressing in the endodontic treatment of teeth with apical periodontitis is necessary.

### Objectives {7}

Our principal objective in this randomized, prospective, double-blinded, controlled clinical trial is to evaluate the effectiveness of endodontic treatments conducted in a single visit with the use of ultrasonic irrigant activation and in two visits with the use of ultrasonic irrigant activation and with and without the use of Ca(OH)2 dressing, in mandibular molars with asymptomatic apical periodontitis regarding periradicular repair during a period of 2 years of clinical, radiographic, and tomographic follow-up. Our secondary objectives are to analyze patients’ postoperative pain levels in periods of 24, 48, and 72 h and 7 days after the completion of a root canal; patient’s satisfaction rate with the treatment that will be performed; and patient’s preference regarding the frequency of clinical visit.

### Trial design {8}

This is a randomized, double-blinded, paralell group, non-inferiority controlled trial.

The trial protocol conforms with the Consolidated Standards of Reporting Trials (CONSORT) Statement. The trial design and protocol adhere to the Standard Protocol Items of the Recommendations for Interventional Trials (SPIRIT) criteria.

**Fig. 1:**
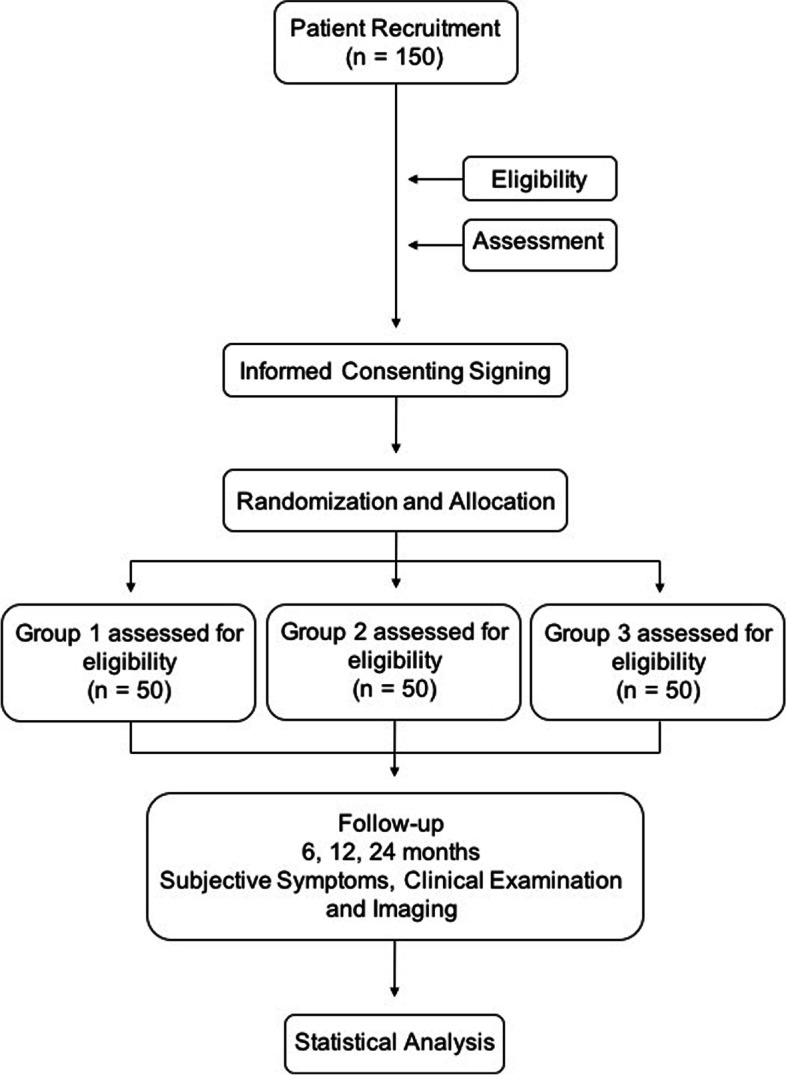
Study flowchart. The trial will evaluate the effectiveness of endodontic treatments conducted in a single visit with the use of ultrasonic irrigant activation and in two visits with the use of ultrasonic irrigant activation and with and without the use of Ca(OH)2 dressing, in mandibular molars with asymptomatic apical periodontitis regarding periradicular repair during a period of 2 years of clinical, radiographic, and tomographic follow-up, based on the periapical index (PAI)

The study aims to compare the efficacy of endodontic treatments conducted in a single visit using ultrasonic irrigant activation and in two visits also using ultrasonic irrigant activation, with and without the use of calcium hydroxide dressing, in lower molar teeth with asymptomatic apical periodontitis (Fig. 1, “Study flowchart”).

After obtaining informed consent, all eligible patients (*n* = 150) will be randomly divided into three groups, according to the clinical approach used during endodontic therapy: in group 1—root canal treatment in a single visit (RCT-SV): endodontic treatment in a single visit using chemomechanical preparation (rotary NiTi instruments, 5.25% NaOCl, 17% ethylene diamine tetraacetic acid (EDTA)) and passive ultrasonic irrigation (PUI), followed by obturation (gutta-percha and epoxy resin-based sealer) and final restoration (composite resin); group 2—root canal treatment in two visits with intracanal dressing (RCT-TVWD): endodontic treatment in two visits using chemomechanical preparation (rotary NiTi instruments, 5.25% NaOCl, 17% EDTA) and PUI, followed by intracanal dressing [Ca(OH)2 powder paste mixed with camphorated paramonochlorophenol (CPMC) and glycerin in 3:1:1 proportion] and temporary restoration (glass ionomer cement (GIC)) for 7 days. After 7 days, an identical chemomechanical preparation on visit 2 was performed, followed by obturation (gutta-percha and epoxy resin-based sealer) and final restoration (composite resin); and group 3—root canal treatment in two visits without intracanal dressing (RCT-TVWOD): endodontic treatment in two visits using chemomechanical preparation (rotary NiTi instruments, 5.25% NaOCl, 17% EDTA) and PUI, followed by temporary restoration with composite resin (in order to avoid contamination of the root canal system) for 7 days, without intracanal dressing. After 7 days, an identical chemomechanical preparation on visit 2 was performed, followed by obturation (gutta-percha and epoxy resin-based sealer) and final restoration (composite resin). The follow-up period will be 24 months, with clinical data collected soon after endodontic therapy. Patients’ preference in choosing the number of clinical visits and the satisfaction rate after completion of treatment will be noted. Patients’ postoperative pain levels will also be recorded in periods of 24, 48, and 72 h and 7 days. Subsequently, clinical findings and long-term follow-up evaluations, with periradicular repair, will be performed by IOPAR at 6 and 12 months and CBCT at 24 months of follow-up.

The primary objective of this study is to evaluate the efficacy of endodontic treatments carried out in a single visit with the use of ultrasonic irrigant activation and in two visits also with the use of ultrasonic irrigant activation and with and without the use of Ca(OH)2 dressing, in lower molar teeth with asymptomatic apical periodontitis. The secondary objective is to carefully investigate the factors influencing the clinical outcomes of a single-visit endodontic treatment of teeth with asymptomatic apical periodontitis and, at the same time, explore the clinical conduct in two visits also with the use of ultrasonic irrigant activation and with the use of intracanal dressing during endodontic therapy of teeth with asymptomatic apical periodontitis.

## Methods: participants, interventions, and outcomes

### Study setting {9}

The study will be conducted in the private offices of two of the co-investigators involved in the study (GMA and VHMC), located in the cities of Itabuna (Bahia) and Goiânia (Goiás), Brazil, respectively. All investigators and examiners are specialists in Endodontics with more than 10 years of clinical experience. They will participate in this randomized clinical trial after receiving adequate training to obtain a comprehensive view of the principles and strategy of the 3 clinical approaches that will be used. A total of 150 adult patients, aged between 18 and 60 years old, who voluntarily seek endodontic treatment in the Brazilian Unified Health System in the cities of Itabuna, in the State of Bahia, and in Goiânia, in the State of Goiás, Brazil. After an assessment of eligibility and informed consent, patients will be randomly assigned to one of 3 clinical study groups. Patients who agree to participate in this study will sign an informed consent form.

### Eligibility criteria {10}

#### Inclusion criteria

To enroll in the study, subjects must (1) be diagnosed with apical periodontitis in lower molar teeth (first or second molars), the periradicular lesion with a diameter between 1 and 5mm analyzed through the real tomographic scale of 1:1; (2) be asymptomatic; (3) spontaneously agree and sign the informed consent form; (4) have an absence of any chronic systemic disease; and (5) be not taking antibiotics and anti-inflammatory drugs.

#### Exclusion criteria

Patients may not participate in the study if they are as follows: (1) teeth with extensive coronary destruction that makes direct restoration with composite resin unfeasible, (2) calcified teeth, (3) teeth with incomplete root formation, (4) teeth with persistent exudation, (5) teeth with anatomical complexities that prevent endodontic treatment in a single visit, (6) teeth recommended for endodontic retreatment, (7) teeth with advanced periodontal pocket, and (8) teeth in which foraminal patency is not obtained.

Patients who present a profile that fits to that described in the inclusion and exclusion criteria will then be referred for evaluation by other examiners (EBPS, LSB, and JRCJr., experts in Endodontics for more than 15 years), for confirmation of diagnosis and treatment planning, if necessary. Patients who do not fit the required profile will be forwarded to the health unit of origin. All the steps of interviews and clinical examination necessary to complete the diagnosis will be carried out by the examiners who will perform the treatment.

Patients with systemic diseases (diabetes, transplants, heart disease, liver failure, and kidney failure) and immunodepressed patients are prohibited during the trial as these conditions can affect the oral functions.

### Who will take informed consent? {26a}

All participants will be given an information sheet including the names and affiliations of the investigators, a description of the study, and its duration. They will have the right to withdraw at any time without giving reasons, ethics committee approvals, and the personal data privacy guarantee. Patients will have unlimited time to read the consent and ask questions. After the period of reflection, during the enrolment visit, the written informed consent will be signed.

### Additional consent provisions for collection and use of participant data and biological specimens {26b}

Not applicable (N/A). There was neither collection nor use of participant data and biological specimens.

## Interventions

### Explanation for the choice of comparators {6b}

Traditionally, root canal treatment in teeth with apical periodontitis is performed in multiple visits, with the use of intracanal dressing between visits, aiming to reduce microorganisms and their by-products from the root canal system prior to filling. However, this type of conventional endodontic treatment has as a disadvantage the inconvenience of requiring patient return (in multiple sessions) for medication changes, until the root canal filling is possible. In the comparison group, the root canal treatment will be performed in a single session, which represents a more objective form of treatment, with the convenience of only one appointment for the patient and a lower operating cost for the professional.

### Intervention description {11a}

In order to treat apical periodontitis, in the first visit, the same endodontic instrumentation protocol will be performed for all teeth randomly divided into the 3 study groups. What will vary will be complementary and additional maneuvers such as the use of ultrasonic activation and intracanal dressing between visits of endodontic instrumentation. All endodontic therapy procedures will be performed under a dental microscope, except for anesthesia and rubber dam placement steps.

#### First clinical appointment

The first visit will include the following clinical protocol depending on the study group: for the 3 groups (G1, G2, and G3): (1) Anesthesia and caries removal. After local anesthesia with articaine hydrochloride and epinephrine tartrate injection (with 1:200,000 adrenaline [Produits Dentaires Pierre Rolland, Merignac, France]), all decayed tissue from the tooth is removed. (2) Isolation and access preparation. The tooth is isolated with a rubber dam and disinfected, and the pulp chamber will be completely unroofed. (3) Initial irrigation with 5 ml of 5.25% NaOCl. (4) Root canal preparation. The #10 C-Pilot file will be used to perform the glide path along the length of the tooth on the radiograph, irrigated with 2 ml of 5.25% NaOCl, followed by rotary instrumentation with #15/.03, #25/.04, and #30/.05 NiTi files, initially in the cervical and middle thirds, after which the working length with the foraminal locator will be performed. Finally, the instrumentation of the apical third will be performed with the same sequence of NiTi rotary files used previously. (5) The canal will be copiously irrigated in three stages using 10ml of 5.25% NaOCl for each of thirds: cervical, middle and apical, totaling 30ml, followed by a final rinse with 10ml of 5.25% NaOCl stirred with the ultrasonic inserts and 10 ml of 17% EDTA stirred also with ultrasonic inserts. The final rinse will be carried out, according to Haapasalo et al. [21], with 10ml of 70% isopropyl alcohol.

##### Group 1—root canal treatment in a single visit (RCT-SV)

(1) The root canals will be filled in the first visit. In this group, a #30/.05 gutta-percha cone and epoxy resin-based sealer with the continuous heat wave technique and a final restoration with Z250 composite resin (3M ESPE, St. Paul, USA) will be used.

##### Group 2—root canal treatment in two visits with intracanal dressing (RCT-TVWD)

(1) The root canals will receive intracanal dressing with Ca(OH)2 (supplied by Farmácia Fórmula & Ação, São Paulo, Brazil), CPMC (Supplied by Farmácia Fórmula & Ação, São Paulo, Brazil), and glycerin (Supplied by Farmácia Fórmula & Ação, São Paulo, Brazil) paste for a period of 7 days. To restrict bacterial regrowth and supply continued disinfection, the root canal will be filled homogeneously to the working length with Ca(OH)2/CPMC/glycerin paste. The tooth will be shielded with glass ionomer cement (GIC) (Vidrion R, SSWhite, Rio de Janeiro, Brazil) as a temporary restoration.

##### Group 3—root canal treatment in two visits without intracanal dressing (RCT-TVWOD)

(1) The root canals will be without intracanal dressing for a period of 7 days, taking into account that the tooth will be restored with composite resin as a temporary restoration (in order to avoid contamination of the root canal system).

#### Second clinical appointment

The second visit will include the following clinical protocol depending on the study group: for the 2 remaining groups (group 2 and group 3): (1) Anesthesia and restorative material removal. After local anesthesia with articaine hydrochloride and epinephrine tartrate injection (with 1:200,000 adrenaline [Produits Dentaires Pierre Rolland, Merignac, France]), all temporary restorative material from the tooth is removed. (2) Isolation and access. The tooth is isolated with a rubber dam and the root canal will be accessed one more time.

##### Group 2

(1) After 7 days, the intracanal dressing will be removed and the root canal will receive a new chemomechanical preparation, identical to the one performed in the first visit, and then it will be filled and permanently restored similarly to group 1.

##### Group 3

(1) After 7 days, the root canal will receive a new chemomechanical preparation, identical to the one performed in the first visit, and then it will be filled and permanently restored similarly to group 1.

### Criteria for discontinuing or modifying allocated interventions {11b}

(1) The presence of serious adverse events that doctors believe should lead to termination of trial participation, such as severe internal or external root resorption and tooth fracture; (2) poor clinical compliance; and (3) withdrawal of consent for study participation by the patient.

### Strategies to improve adherence to interventions {11c}

During the study, patients will receive text messages and phone calls to remind them of their appointments for treatment and follow-up visits and to answer the questionnaires.

### Relevant concomitant care permitted or prohibited during the trial {11d}

Patients are prohibited to take any anti-inflammatory or antibiotic medication during their participation at this research study.

### Provisions for post-trial care {30}

At the end of the study, patients will be proposed to continue their long-term oral follow-up at the Brazilian Unified Health System in the cities of Itabuna, in the State of Bahia, and in Goiânia, in the State of Goiás, Brazil.

### Outcomes {12}

All the steps of clinical examination will be carried out by the two examiners responsible for the study. Imaging evaluation will be done by other 2 specialists in Endodontics and 1 radiologist. All are with at least 15 years of experience.

Assessments will also be carried out at 6 (IOPAR), 12 (IOPAR), and 24 (CBCT) months for analysis of the tissue repair process. The therapeutic effect evaluation criteria are the following: (1) success: after RCT, the tooth should be asymptomatic, without fistula, without pain on percussion and palpation tests, without edema and in normal chewing function, radiographically in the process of periapical lesion regression from the first assessment (time point) at 06 months, and fully repaired after 2 years with confirmation through CBCT; and (2) failure: after RCT, the tooth is symptomatic, with symptoms of apical infection such as pain and fistula. The radiographic examination demonstrates the absence of decreased apical lesion or an enlarged apical lesion [22, 23].

#### Primary outcome measures

##### Healing of periradicular lesions by radiographic findings according to periapical index (PAI)

The periapical index (PAI) is a structured scoring system for categorization of radiographic features of apical periodontitis. It is based on a visual scale of periapical periodontitis severity and was built upon a classical study of histological-radiological correlations. It is a 5-point ordinal scale as listed below: (1) normal periapical structures, (2) small changes in bone structure with no demineralization, (3) changes in bone structure with some diffuse demineralization, (4) apical periodontitis with a well-defined radiolucent area, and (5) severe apical periodontitis, with exacerbating features [22, 23].

#### Secondary outcome measures

##### Patient’s postoperative pain using a questionnaire

**Fig. 2:**
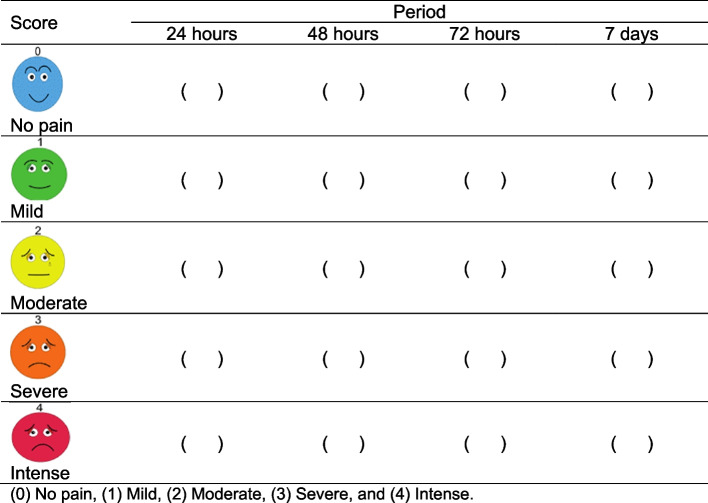
Patient’s postoperative pain—pain scale (VAS) questionnaire

Patients will answer a questionnaire for the analysis of postoperative pain, through the analog pain scale, in an interval of 24h, 48h, and 72h and 7 days: (0) no pain, (1) mild, (2) moderate, (3) severe, and (4) intense (Fig. 2).

##### Patient’s preference regarding the number of clinical visits using a questionnaire

**Fig. 3:**
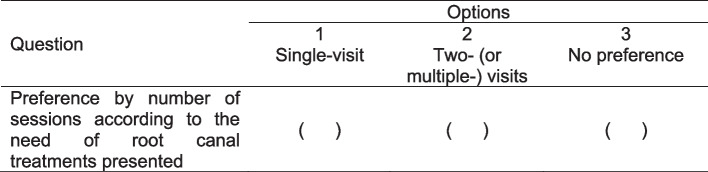
Questionnaire on the patient’s preference regarding the number of clinical visits to be planned for the conduction of endodontic treatment

For the data on the patient’s preference regarding the number of clinical visits to be planned for the conduction of endodontic treatment, patients will answer their preferences according to hypothetical conditions described in a previously delivered questionnaire: (1) single visit, (2) two (or multiple) visits, and (3) no preference (Fig. 3).

##### Patient’s post-treatment satisfaction using a questionnaire

**Fig. 4:**
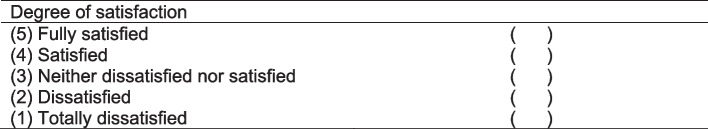
Patient’s post-treatment satisfaction degree questionnaire

A post-treatment satisfaction questionnaire will be applied to patients undergoing clinical interventions in a single visit and multiple visits, using the following criteria: (5) fully satisfied, (4) satisfied, (3) neither dissatisfied nor satisfied, (2) dissatisfied, and (1) totally dissatisfied (Fig. 4).

### Participant timeline {13}

The study timeline is summarized in Table 1.

**Table 1:**
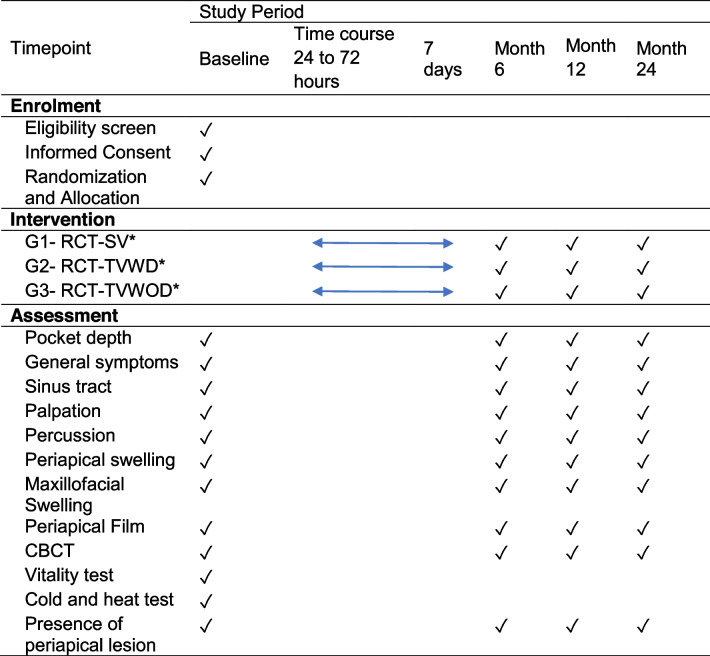
Study timeline: enrolment, interventions, and assessments

*Group 1-RCT-SV, root canal treatment in a single visit; *group 2-RCT-TVWD*, root canal treatment in two visits with intracanal dressing; *group 3-RCT-TVWOD*, root canal treatment in two visits without intracanal dressing

### Sample size *{14}*

The determination of the sample size will be performed sequentially, with a minimum of 210 statistical units (teeth) to be recruited in the first stage, and the second stage of recruitment will be performed if the selection is insufficient to clinically and radiographically identify valid cases. This sampling will also allow the adjustment of the sample size for the intra-subject correlation of statistical units (teeth). In the first stage, 150 adults aged 18 to 60 years, with at least one tooth diagnosed with asymptomatic apical periodontitis and periradicular lesion with a diameter between 1 and 5 mm, will be recruited.

## Sample size and statistical methods

### Sample size details

The required sample size was calculated based on the preliminary outcome parameter of success, in a sequential approach, with each clinical case (tooth) serving as the statistical unit. Clinical studies that allow us to estimate, by means of CBCT, the repair index (complete absence of periapical radiolucency), therefore the success, after endodontic treatment of teeth with apical periodontitis and periradicular lesion, state that it is necessary to have a follow-up of at least 2 years to determine the success or failure of the treatment [22, 23]. After the first year of follow-up, post-endodontic treatment, it is still possible to observe the presence of periapical radiolucency in 52 to 84% of cases [1, 24, 25]. The sample will consist of 210 mandibular molars (first or second molars), based on statistical sample calculation, considering a power of 80%, an alpha value of 0.05, and an effect size of 20%. All patient characteristics at the beginning of treatment, such as age, gender, lesion size, among others, will be considered in the statistical analysis, in order to confirm the randomization of groups and the possible confounding effect of these characteristics.

### Recruitment {15}

For achieving adequate participant enrolment to reach the target sample size, all new patients meeting eligibility criteria will be given the opportunity to participate in the study. A close coordination is made between the private offices of both co-investigators involved in the study (GMA and VHMC) to identify and refer adequately potential participants.

## Assignment of interventions: allocation

### Sequence generation {16a}

Assignments will be prepared by the statistician author of this study (TLA) prior to the trial start. A total of 150 patients (210 teeth) will be recruited and divided into 3 groups at a ratio of 1:1:1, namely, the RCT in a single visit, RCT in two visits with intracanal dressing, and RCT in two visits without intracanal dressing.

Randomization and allocation will be performed before all the clinical procedures.

First, a list of numbers from 1 to 210 will be created, each of the number represents one participant, then the list of numbers was assigned randomly following simple randomization procedures, by using Excel 2019 (Microsoft, Redmond, WA, USA) into three groups of 50 participants (70 teeth) each. Eventually, two columns in the spreadsheet are used, the first column contains the number of the participant (teeth) and the second contains the group to which the participant (teeth) is allocated, determined solely by the software.

### Concealment mechanism {16b}

Each number and the group to which a number is allocated will be concealed in sequentially numbered, opaque, sealed, and stapled envelopes. Only investigators can open the envelope, at the moment of intervention, to check the group to which the number is allocated, and perform the interventions according to the instructions of this study.

### Implementation {16c}

The randomization list is implemented in the software by the study statistician (TLA). Access to the randomization list is limited to the study investigators.

## Assignment of interventions: blinding

### Who will be blinded {17a}

As a double-blinded trial, the patients and outcome evaluators will be blinded to the group assignment until the completion of the study. As dentists cannot be blinded to treatment allocation due to the notable differences in the treatment methods, they will not be allowed to discuss the type of intervention with either patients or outcome evaluators.

### Procedure for unblinding if needed {17b}

We do not anticipate any requirement for unblinding, but if required, the principal investigator or the data manager will have access to group allocations and any unblinding will be reported.

## Data collection and management

### Plans for assessment and collection of outcomes {18a}

Data associated with this study will be collected in the standardized case report forms (CRF) for the outcome analysis, and a specific supervisor will be responsible for reviewing the integrity, accuracy, and consistency of the data. To ensure the accuracy of data entry, three investigators (two endodontists and 1 radiologist) will be responsible for entering the data independently and data query forms (DQF) will be resolved by tracing the source data.

A questionnaire to score the patient postoperative pain levels will be administered by two investigators in periods of 24, 48, and 72 h and 7 days after the completion of a root canal treatment. Questionnaires of patient satisfaction rate with regard to the treatment and the patient preference in relation to the frequency of clinical visits will also be performed. The secondary outcomes will be obtained from self-administered questionnaires completed immediately after the completion of a root canal. All the data will be registered in document clouds (in a specific Microsoft 365 OneDrive institutional account files), and only authorized personnel will have access. Data monitoring will be independent from the investigators and competing interests.

### Plans to promote participant retention and complete follow-up {18b}

A patient will be considered as lost-to-follow-up if no contact can be made during 24 months, after an active search from the two investigators. Participants will receive no financial compensation but they will be given the root canal and the restorative treatments of the teeth.

### Data management {19}

Data are entered anonymously. Data collection will be monitored by a clinical research assistant. When requested, the two investigators will clarify the data. Data management is under the responsibility of the University of Brasilia.

The Data Management Coordinating Center will oversee the intra-study data-sharing process, with the involvement of the Data Management Subcommittee. Only the principal investigator will be given access to all the data with a special password. Other project investigators will have direct access to their own site’s data sets and other sites’ data by request. To ensure confidentiality, data dispersed to project team members will be blinded to any identifying participant information.

### Confidentiality {27}

The final trial dataset will be available to the clinical research assistants, data managers, and statisticians, subject to professional secrecy. Data are anonymous.

### Plans for collection, laboratory evaluation, and storage of biological specimens for genetic or molecular analysis in this trial/future use {33}

N/A. No collection.

## Statistical methods

### Statistical methods for primary and secondary outcomes {20a}

The data will be analyzed using SPSS 25.0 software (IBM Co., Armonk, NY, USA).

#### Baseline analysis

The three groups will be similar in terms of disease type and periapical lesion size.

#### Main outcome analysis

The inter-group comparison of success rates will be analyzed using the Kruskal-Wallis test at 5% of the level of significance. For the missing data, the last observation carried forward (LOCF) method will be adopted to fill the validity analysis.

#### Secondary outcome analysis

Intra-group and inter-group comparisons for analysis of postoperative pain and post-treatment satisfaction will be performed by Friedman and Kruskal-Wallis tests, respectively. Inter-group comparisons for preferences regarding to the number of clinical visits will be performed by the chi-square test. All tests will be performed at 5% of the level of significance.

### Interim analyses {21b}

N/A. No interim analyses are planned.

### Methods for additional analyses (e.g., subgroup analyses) {20b}

N/A. No additional analyses are planned.

### Methods in analysis to handle protocol non-adherence and any statistical methods to handle missing data {20c}

Protocol violations after randomization will be listed in the clinical study report, tabulated by subject and recruitment site. We will perform intention-to-treat analyses with a “missing=failure” strategy to the management of missing data.

Sensitivity analyses will be performed for the missing data management: multiple imputation, available data, and maximum bias.

### Plans to give access to the full protocol, participant-level data, and statistical code {31c}

The full protocol, participant-level data, and statistical code are available upon request and after a contract has been put in place to ensure, among other things, that the recipient complies with the GDPR.

## Oversight and monitoring

### Composition of the coordinating center and trial steering committee {5d}

The steering committee is made up of the following people: Dr Gustavo M. Almeida (chairman, principal investigator), Dr Vitor Hugo M. Carvalho (clinical investigator), Dr André F. Leite (radiologist and responsible for the center of Brasilia), Dr Erica L. Queiroz, Dr Ana Paula D. Ribeiro, Dr Jacy R. Carvalho-Junior (methodologists), and Dr Tien Li An (statistician). This committee checks ethics. With the Center for Methodology and Data Management, this committee checks also the status of the research, possible problems, and available results. It decides on any relevant modification of the protocol necessary for the continuation of the research. It may propose to extend or interrupt the research.

### Composition of the data monitoring committee, its role, and reporting structure {21a}

The establishment of a data monitoring committee is not necessary for this study, which does not entail any particular risk a priori for the participants.

### Adverse event reporting and harms {22}

Adverse events that may occur will be monitored by the investigators and a research assistant. All information regarding adverse events during the study will be recorded in detail, including symptoms, signs, onset time, and severity. Some possible adverse events that may be attributed to RCT include reinfection of the root canal, flare-up, root and coronary fracture, and discomfort to chewing.

### Frequency and plans for auditing trial conduct {23}

In the context of the data monitoring plan, a clinical research assistant mandated by the responsible for the center of Brasilia will visit each investigating center on a regular basis, during the implementation of the research, one or more times during the research according to the rhythm of the inclusions and at the end of the research. An audit can be conducted any time at the request of the responsible for the center of Brasilia and independent from the investigators, but also at the request of the competent health authority.

### Plans for communicating important protocol amendments to relevant parties (e.g., trial participants, ethical committees) {25}

The protocol has been approved by the Medical Ethics Committee of the University of Brasilia CAAE protocol: 87963117.3.0000.0030 and registered at ClinicalTrials.gov. NCT05256667.

Any important protocol amendment must obtain, prior to its implementation, a favorable opinion from a Medical Ethics Committee of the University of Brasilia (Comitê de Ética em pesquisa da Universidade de Brasília [CEPE]). All modifications to the protocol should be brought to the attention of all investigators participating in the research. Any modification that modifies the coverage of participants or the benefits, risks, and constraints of the research is the subject of a new information sheet and a new consent form. Any amendments to the protocol will be reviewed and approved. Written informed consent will be obtained from all patients.

### Dissemination plans {31a}

Results of the trial will be communicated to the participants through a brochure that will be sent at the patient’s home. They will be also submitted to national and international journals for publication.

Results of the trial will be communicated to the participants upon request to the investigators.

## Discussion

Endodontic treatment consists of a type of therapy of considerable complexity due to factors such as the anatomical variety of the root canal system, the presence of curvature of the main root canal, branches of other canals, isthmuses, areas inaccessible to the endodontic instrument [5, 7], and the dentin permeability itself, associated with the possibility of infection of this system [9, 15]. These anatomo-structural characteristics of the root canal system, accompanied by an infectious process, make it necessary to develop techniques that allow for a more predictable cleaning and disinfection of this environment of difficult visibility and access [9]. Cases of apical periodontitis can often bring additional challenges to professionals, due to the presence of periradicular lesion, which characterizes a high level of microbial contamination, in the form of bacterial biofilm, requiring a highly efficient chemical and mechanical action that is able to eliminate the greatest possible number of microorganisms from the root canal system, allowing the organism to respond favorably in the tissue healing and repair process [2]. Inappropriate planning or management of endodontic treatment of a tooth with asymptomatic apical periodontitis can lead to a flare-up, the need for re-intervention, or retreatment [3]. Thus, the use of chemomechanical root canal preparation protocols through engine-driven rotary instrumentation with nickel-titanium (NITi) instruments, which provide faster preparation, associated with the use of irrigating solutions, activated by ultrasound, and intracanal medication, both with high antibacterial potential, is increasingly suggested for the treatment of such pathology, with the objective of promoting a level of disinfection that allows healing (the repair of periradicular tissues) and the absence of postoperative symptoms [26, 27]. However, the option for endodontic treatment in a single visit, where intracanal medication is not used, for a minimum period of 7 days, between visits, is questioned by a considerable number of studies [13–15], often for fear that remaining bacteria after chemomechanical preparation may cause some kind of injury and that the patient may present postoperative pain [17–19]. Obturating a tooth with asymptomatic apical periodontitis in a single visit characterized as an unreliable or unsafe procedure mainly in results of in vivo studies in an animal model [13, 14] or in in vitro studies [15], although the systematic reviews [16–18] and randomized clinical trials [1, 19, 20] show that the results are similar for postoperative pain and cure rate when endodontic treatments conducted in a single visit and in multiple visits are compared. These studies emphasize that the criteria used during chemomechanical preparation are considered more efficient when associated with ultrasound during endodontic treatment. By using irrigating solutions with greater antimicrobial potential associated with methods that promote the agitation of these substances inside the main canal for longer periods of time and with greater amounts of solution, it allows them to reach dentinal tubules or regions further away from the main canal and that ultrasonic cavitation promotes a disruption of the bacterial biofilm, promoting superior clinical results [7] and, consequently, increasing the acceptance of the single visit as a form of treatment for asymptomatic apical periodontitis among professionals. Furthermore, the determination of chemomechanical preparation protocols, based on criteria that enhance the antimicrobial action of irrigating solutions, would allow the standardization of these protocols for scientific purposes, making comparative studies more reliable [16]. Although the successful elimination of bacteria from the root canal system remains the most important therapeutic goal in Endodontics [2], there is no consensus on the most effective clinical approach, raising doubts about what would be the best treatment model to be followed [1]. The present study aims to compare a single-visit endodontic treatment with a two-visit treatment in cases of asymptomatic apical periodontitis using four parameters for outcome analysis: first, clinical success through periradicular lesion repair. It is very important that the clinician knows the time needed to verify the complete repair of a periradicular lesion in a case of apical periodontitis. This knowledge will allow the professional, at the end of the treatment, to guide the patient about the healing process, as well as allow a more objective assessment of a treatment performed by another professional, reducing misdiagnoses of failure and subsequent retreatment proposals. Periapical radiography is the most commonly used method to assess the cure rate in apical periodontitis [1, 13, 16]; however, the two-dimensional image does not represent a real evaluation because it masks results [24], so the proposal is also to evaluate the results through CBCT, comparing with periapical radiography before and after 2 years of follow-up. Second, postoperative pain will be assessed during periods of 24, 48, and 72 h and 7 days. Postoperative pain to endodontic treatment may be related to several factors such as host response to treatment, microbial pathogenicity, or extrusion of debris beyond the foramen caused by inadequate instrumentation [19]. Third, subjective results will be evaluated, that is, those reported by patients. This is relevant, as it brings the subjective perception that the patient had or has about the endodontic treatment and confirms or not the common sense that endodontic treatment and pain are synonymous, promoting behavior changes on the patient’s future attitudes in the next visits when the need arises, to perform a new endodontic treatment. Finally, the study will assess the patient’s satisfaction rate with the treatment performed. This is relevant, as the patient will be able to assess the experience of undergoing endodontic treatment and report their degree of satisfaction after completion of the treatment. Despite all the objectives presented, this study also has limitations. Firstly, the instrumentation and irrigation protocol used in this study, despite being within the best parameters of current endodontic therapy [9, 26, 27], has variations in the time of ultrasound use during cavitation and also in the amount of irrigant made of 5.25% NaOCl with a volume higher than those tested in other studies. Even though group 3 will not have intracanal medication placed, both groups 2 and 3 will have the same number of days between visits in order to avoid confounding variables that could interfere in the study outcome results. Although there are studies in the form of systematic reviews [16–18] and randomized clinical studies [1, 19, 20], showing similar results, regarding the success rate and postoperative pain in cases of endodontic treatments completed in a single visit and in multiple visits in teeth with apical periodontitis, there is no consensus on the instrumentation and irrigation protocols to be used. The same thing happens in vitro studies [15] and in vivo studies in an animal model [13, 14], and there is also no consensus regarding the instrumentation and irrigation protocols. In most of these studies, irrigation protocols describe the use of NaOCl solutions with active chlorine concentrations less than 5.25% [19, 27]. Systematic reviews criticize the volume and time that the irrigating solution remains inside the root canal system [16, 17]. Furthermore, some studies do not use ultrasound as an irrigation intensifying agent [7, 13] and still do not specify the volume of the chemical substance used during treatment [4]. This variety of protocols promotes different results in the studies and, consequently, divergences of opinions and clinical approaches. Secondly, in clinical studies, there is the difficulty of following the patient for long periods after the end of treatment [1], which creates the need to think of special approaches to try to ensure the patient’s permanence in the study, such as offering free oral health promotion actions, for example. Thirdly, the sample size is also considered a complicating factor, since the numbers used in the studies found are quite different, which points to questionable results [14]. Lastly, this study will be conducted in lower molar teeth, which have more complex anatomy, are more affected by periodontal disease, and also because they are the most standardized teeth in randomized clinical trials [28].

## Trial status

This trial is registered at ClinicalTrials.gov, NCT05256667. This report is based on protocol version 1.0. Recruiting started in August 2021 and the estimated end date is December 2024.

## Data Availability

The datasets analyzed during the current study are available from the corresponding author on reasonable request.

## Abbreviations

Ca(OH)2: Calcium hydroxide
IOPAR: Intraoral periapical radiograph
CBCT: Cone beam computed tomography
CMCP: Camphorated paramonochlorophenol
EDTA: Ethylene diamine tetraacetic acid
NaOCl: Sodium hypochlorite
RCT: Root canal treatment
RCT-SV: Root canal treatment in a single visit
RCT-TVWD: Root canal treatment in two visits with intracanal dressing
RCT-TVWOD: Root canal treatment in two visits without intracanal dressing
